# COVID-19 vaccination hesitancy in Australia: a public health issue

**DOI:** 10.3389/fpubh.2024.1282986

**Published:** 2024-01-18

**Authors:** Minh-Hoang Tran

**Affiliations:** NTT Hi-Tech Institute, Nguyen Tat Thanh University, Ho Chi Minh City, Vietnam

**Keywords:** COVID-19, COVID-19 vaccines, vaccination hesitancy, public health, Australia

## 1 Introduction

Since the first outbreak in December 2019 in Wuhan, China, the highly contagious Coronavirus Disease 2019 (COVID-19, caused by the SARS-CoV-2 virus) spread quickly worldwide and became a pandemic (1). In Australia, over 4 million cases were identified, resulting in ~6,000 deaths (2). The burdens to the healthcare systems and people's wellbeing are also increasing due to the negative outcomes of post-COVID conditions (3).

In confronting this pandemic, COVID-19 vaccines were rapidly developed to mitigate the negative health impact of SARS-CoV-2, primarily by reducing hospitalization and mortality rates (4). The subsequent vaccination rollouts have shown remarkable effectiveness with decreased hospital admission rates, serious illness, and death. Despite these encouraging results, vaccination hesitancy, referring to the refusal or delay in accepting vaccines despite available supplies and services (5), was posing significant issues to COVID-19 control strategies in many countries. While this had already emerged long before, it has become a critical challenge to establishing herd immunity (6), increasing the risks for new outbreaks and negative outcomes for vulnerable populations (i.e., immunocompromised or comorbid patients).

Optimistically, in Australia, data from the Vaccine Hesitancy Tracker signaled a slowing fall from 30 to 10% in the proportions of reluctant adults (7). However, with the necessity of booster doses to enhance protections against emerging variants, around 25% of Australians were hesitant (7). This can lead to adverse consequences due to COVID-19 burdens. Vaccine hesitancy may also disproportionately influence underrepresented and minority groups (i.e., Aboriginal and Torres Strait Islander people) (8), whose vaccination rates are lower than the general population (9). More concerningly, unpublished data recorded only 48% of intended vaccination in Australian pregnant women (10), one of the vulnerable populations to COVID-19, which increases their risks of hospitalization, critical illness, or even mortality. Since hesitating to get vaccinated could soar overall rates of hospitalization, the resultant cost of hospital care for COVID-19 can also put Australian public hospitals in financial crisis (11), as one preventable case is estimated to save 68,000–104,000 AUD (12). While these data implicate possible impacts of vaccine hesitancy, there is a limitation of lacking confirmed evidence from population-based studies, which can diminish the importance and underestimate the severity of this concerning affair. Given the potential burdens of COVID-19 vaccination hesitancy, this should be regarded as a public health issue in Australia.

## 2 Causes of vaccination hesitancy

Certain works have aimed to identify the causes of this condition, especially regarding the COVID-19 pandemic. There were two most common models that characterize the determinants of vaccine hesitancy, the 3Cs model (5) and its extended version, the 5Cs model (13). The former—including confidence, complacency, and convenience—addresses building trust in vaccines, improving accessibility, and combating the perception that vaccine-preventable diseases are no longer a threat (5). The latter replaces convenience with constraints and added two more factors of risk calculation and collective responsibility (13). One thing to note here is all the listed determinants can imply both risk factors and enablers of vaccine hesitancy depending on the way people perceive these aspects. In the Australian setting, the main model-based drivers of COVID-19 vaccination hesitancy are confidence and complacency (14).

### 2.1 Confidence

Confidence is defined as “trust in (i) the effectiveness and safety of vaccines, (ii) the system that delivers them, including the reliability and competence of the health services and health professionals, and (iii) the motivations of policy-makers who decide on the need of vaccines” (15). In most cases, confidence is deemed as a risk factor for vaccine hesitancy, as without trust in the three listed aspects, people are likely to develop negative attitudes toward vaccination. This was partially confirmed with a relatively strong association between trust barriers and vaccine hesitancy (OR = 1.70, 95% CI: 1.11–2.60) (16). However, trust is a matter of gaining, not being granted. Amid the COVID-19 pandemic, there have been various misleading contents (i.e., rumors, misinformation, conspiracy theories) worldwide (17), including in Australia (18, 19). Despite no supporting evidence, these fear-based contents can drive people toward negative attitudes (20), contributing to the vaccine hesitancy. The critical point here is to identify where these false claims stem from. Tracing back to when new findings of COVID-19 vaccines' safety were published, non-expert viewers were very likely to be afraid of the jabs due to some reports about unverified serious adverse effects of the available vaccines (18). Without proper perception, the general population would be misled by self-serving conspiracists exploiting the science of COVID-19 vaccines (21). Therefore, the root cause of lacking people's trust is that governments, in Australia or worldwide, need effective measures to address the spread of misinformation about COVID-19 vaccination.

### 2.2 Complacency

Complacency “exists where perceived risks of vaccine-preventable diseases are low and vaccination is not deemed a necessary preventive action” (15). Similar to confidence, in Australia, complacency is majorly considered a risk factor for vaccine hesitancy (14), possibly due to prospective results in the first year of the pandemic (i.e., modest disease burden, relatively low level of cases and deaths) (22). A study in 2021 partly verified a strong relationship between attitudes of low risk from COVID-19 and vaccine hesitancy (OR = 2.02, 95% CI: 1.58–2.59) (16). It is reasonable for people who do not feel the threats from COVID-19 to hesitate or even neglect vaccination (23). However, the fact of low infection rates and risks at the start of the pandemic in Australia cannot conclude the mild nature of COVID-19. Various factors were attributable to these temporary outcomes, including modifiable (i.e., virus variants, face mask, social distancing, lock-down) and unmodifiable ones (i.e., geographic isolation). Given the proper initial prevention measures of the Australian government (24), people may not realize how contagious new COVID-19 variants might be and how important getting booster vaccination is. During the Omicron emergence, it was critical to identify why people were not aware of the booster jabs' necessity. Omicron is an extremely contagious variant (25), causing the daily new cases to rocket over a few months (26). Nevertheless, many people thought of this as a mild infection (25), without knowing the potential risks due to the widespread of Omicron. If they had fully perceived the risks of post-COVID conditions (27) or the emergence of more dangerous variants following Omicron (28), perhaps they would not have hesitated for any booster dose of COVID-19 vaccines. Thus, the potential root cause of complacency is the lack of providing updated and reliable knowledge in a widely accessible way to keep the general population informed of their decisions.

### 2.3 Limitations of 3Cs and 5Cs models

Despite being valuable models in analyzing vaccine hesitancy, both the 3Cs and 5Cs models have a limitation in predictive validity (13), meaning they were not validated to predict the vaccination behavior in the future. Additionally, as these models were developed in high-resource settings, they might not be completely applicable to underrepresented settings (i.e., Aboriginal and Torres Strait Islander). Apart from the model-based determinants, the relationships between the root cause of the factors and the issue of vaccine hesitancy have not been thoroughly evaluated in confirmatory studies.

## 3 Discussion

### 3.1 Prevention level and strategy type

Overall, the main causes of both confidence and complacency in Australia can be generalized to a nationwide information crisis, where evidence-based information is not as favored as false claims and irrational rumors. Based on this, the most appropriate prevention level is primordial, which aims to establish and maintain environmental, economic, social, and behavioral conditions that “minimize hazards to health” (29). The first reason is, as the initial stage, the primordial level better prevents the information crisis, as it seems easier to control rumors when they are not spread away. Following that, reliable information under expert interpretation could be advocated and accessed widely by the general public, leaving very limited chance for misleading news to appear. Another reason is that primordial prevention primarily targets the total population (29), allowing a rapid and broad effect with minimal costs. Although this would also mean high-risk groups may not be specifically targeted, and individual-level effectiveness in these groups is less likely to be achieved, given the strengths of primordial prevention, this seems to be the most prominent approach in the Australian setting.

Following primordial prevention, governmental policy-based strategies should be a reasonable approach in this case. With a widely-orientated policy, the effectiveness of primordial interventions could be maximized, i.e., through enforcing a strict and heavily-fined ban on spreading unverified news about COVID-19 vaccines. While this sounds extreme and restricts the freedom of speech, its benefits should outweigh the risks, especially when Australia was sometimes on the verge of uncontrolled outbreaks with exploding daily cases (30).

### 3.2 Incorporation into guiding principles

Within the context of COVID-19 vaccination hesitancy in Australia, firstly, it is important to thoroughly understand how exactly all the known determinants of vaccine hesitancy affect individual's behaviors. This will lead to more precise interventional policies that focus on the root causes of the issue (31). Additionally, as policies can have a broad effect on the population, practice based on sound evidence should also be implemented. In this way, the perspectives of stakeholders could be incorporated to address the needs and extent of effectiveness of the policy (31).

Moreover, peoples' and governments' responses to COVID-19 and other infectious diseases, e.g., influenza, are different. The unique effect of COVID-19 comes from a combination of lockdown/quarantine, massive rates of hospitalization and mortality within a short period, and various variants with various virulences. Therefore, while the strategies to prevent vaccination hesitancy in other vaccine-preventable diseases can be potentially applied for COVID-19, the principles and actual policies should have been specifically tailored for this global pandemic.

## Author contributions

M-HT: Conceptualization, Writing – original draft, Writing – review & editing.
